# Cecal volvulus after open right hepatectomy

**DOI:** 10.1093/jscr/rjae836

**Published:** 2025-02-12

**Authors:** Lasse R Jensen, Niclas Dohrn, Luit Penninga

**Affiliations:** Department of Surgery and Transplantation, Rigshospitalet, Copenhagen University Hospital, Copenhagen, Denmark; Department of Surgery and Transplantation, Rigshospitalet, Copenhagen University Hospital, Copenhagen, Denmark; Department of Surgery and Transplantation, Rigshospitalet, Copenhagen University Hospital, Copenhagen, Denmark; Institute of Clinical Medicine, University of Copenhagen, Blegdamsvej 3b, 2200 Copenhagen N, Denmark

**Keywords:** volvulus, hepatectomy, postoperative, emergency

## Abstract

Cecal volvulus, a rare cause of postoperative bowel obstruction, involves the twisting of the cecum and its mesentery, leading to the risk of ischemia. We present a case of cecal volvulus following open right hepatectomy in a patient in her 70s with no prior abdominal surgeries. On postoperative day six, after developing abdominal distension and pain, a CT scan revealed bowel obstruction and a cecal volvulus was suspected. Emergency laparotomy confirmed cecal volvulus with a highly mobile cecum, necessitating right hemicolectomy with end-ileostomy due to sepsis. The patient’s recovery was prolonged due to complications, including postoperative paralysis and fascial dehiscence. To our knowledge, this is the first documented case of cecal volvulus following right hepatectomy, likely due to altered anatomy and postoperative paralysis. This case underscores the importance of considering cecal volvulus in postoperative obstruction cases, especially following surgeries affecting right-sided visceral structures.

## Introduction

Volvulus is a condition where a bowel loop and its mesentery twist around a fixed point, leading to mechanical bowel obstruction and potential ischemia, gangrene, and perforation. Cecal volvulus constitutes 10%–40% of all colonic volvulus cases [1]. Cecal volvulus presumably has a multifactorial etiology, with adhesions, distal obstruction, pelvic masses, pregnancy, colonoscopy, and recent surgical manipulation as proposed contributing factors [2, 3]. Furthermore, autopsy studies indicate that ⁓10%–25% of the population have a sufficiently mobile cecum and ascending colon, allowing them to develop a volvulus [4]. Patients can present with varying symptoms ranging from intermittent abdominal pain episodes to a severe acute abdomen. Computed tomography (CT) scan is diagnostic in 90% of cases, while the remaining 10% are diagnosed with a contrast enema or at surgical exploration [5].

## Case report

**Figure 1:**
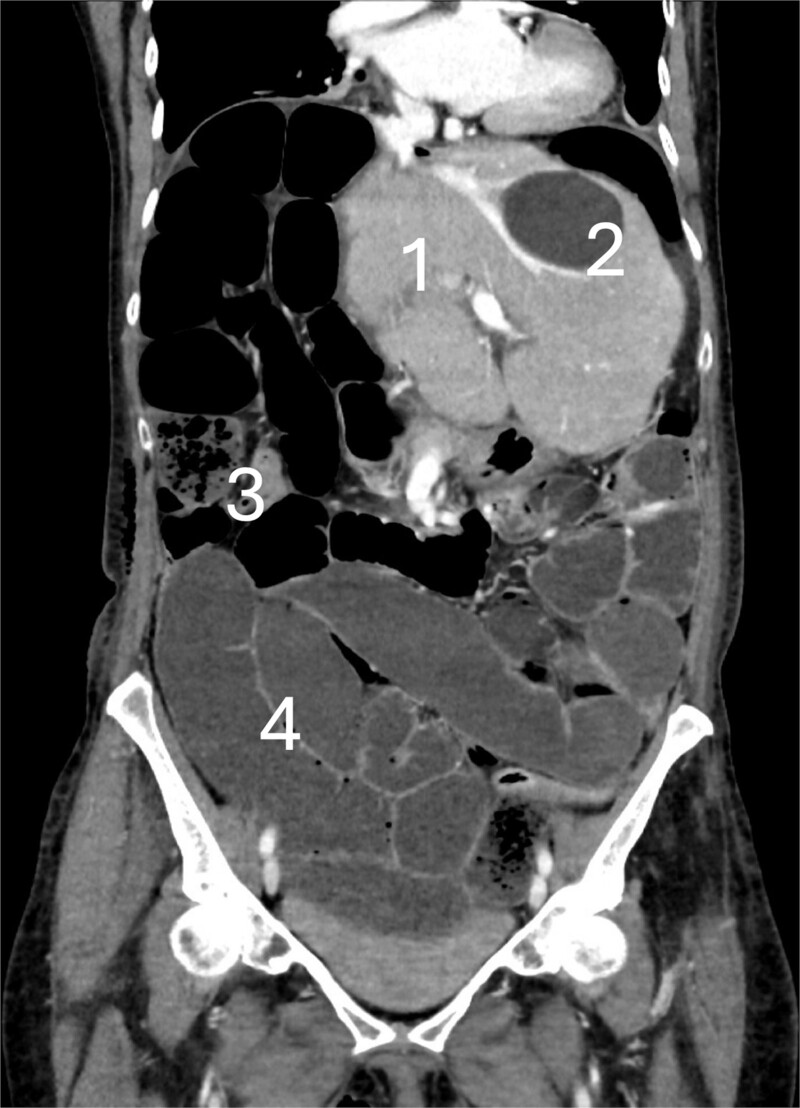
CT scan at the 6th postoperative day after hepatectomy showing cecal volvulus with: (1) Remnant liver, (2) benign liver cyst, (3) small bowel feces sign, and (4) fluid-filled dilated small bowel.

**Figure 2:**
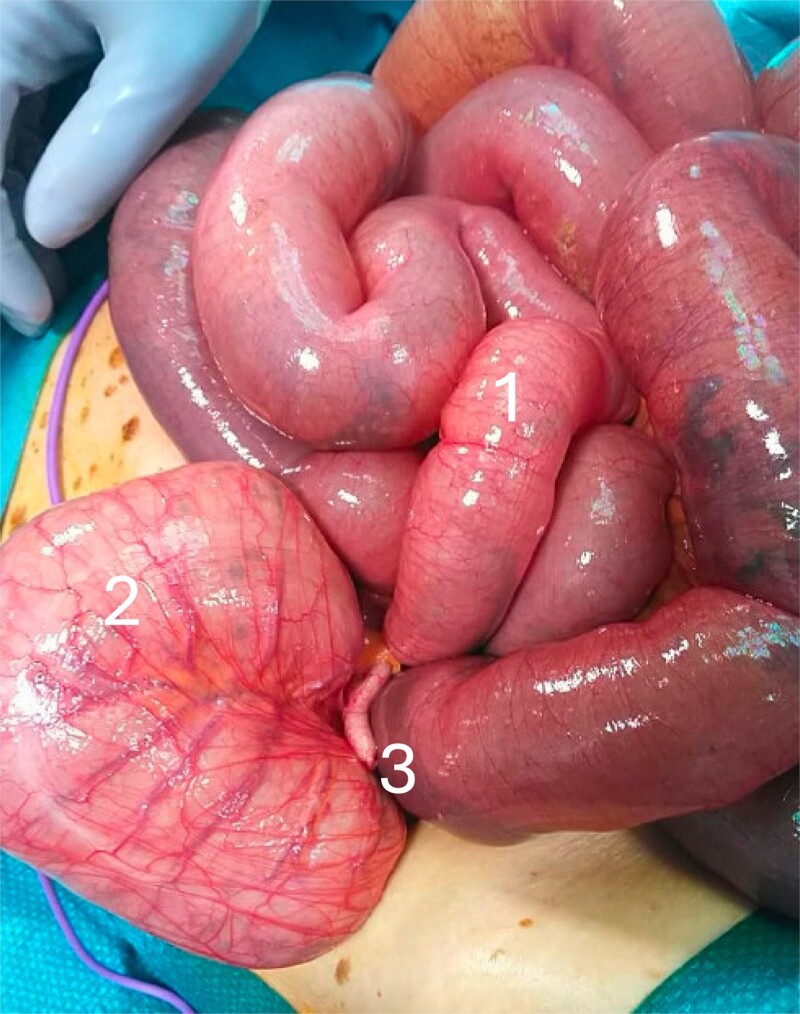
Intraoperative photo showing the cecal volvulus with: (1) distended small bowel due to mechanical bowel obstruction, (2) rotated and distended cecum, and (3) appendix.

We report on a patient in her 70s with no history of abdominal surgeries, who underwent an elective open right hepatectomy for a large (11 cm) cholangiocarcinoma without signs of extrahepatic metastases. Due to an extensive centrally located tumor, an open resection was performed. The tumor was staged as pT1b. No direct mobilization of the colon was performed. On the fourth postoperative day (POD), a CT scan was conducted due to abdominal distension and absence of defecation, indicating signs of paralysis. On POD 6, the CT scan was repeated because of worsening abdominal pain and lack of clinical improvement, showing mechanical small bowel obstruction at the terminal ileum, and suspicion of cecal volvulus (Fig. 1). The patient underwent emergency laparotomy showing a cecal volvulus (Fig. 2). Intraoperatively, a pronounced mobile cecum was identified, and a right hemicolectomy was performed. Due to sepsis and the requirement for noradrenaline, a primary anastomosis was not feasible, so an end-ileostomy was performed. The patient was transferred to the Intensive Care Unit (ICU) postoperatively. During the postoperative course the patient experienced prolonged paralysis, surgical site infection, bacterial contaminated ascites, and fascial dehiscence. The patient was discharged at POD 42. The patient was doing fine 3 months after surgery.

## Discussion

Cecal volvulus is a very seldom postoperative complication, with few cases reported after left colectomy, cholecystectomy, gastric resection, incarcerated femoral hernia repair, appendectomy, various laparoscopic procedures, kidney transplantation, and nephrectomy [6]. To our knowledge, this is the first case of cecal volvulus after hepatectomy. Any surgical intervention that necessitates medial visceral rotation or interferes with the fusion plane between the colon and the lateral peritoneum might provide the necessary mobility for a cecal volvulus to develop. In this case, the right colon or flexure were not directly mobilized intraoperatively, however, the altered anatomy following the hepatectomy might have contributed to the volvulus. Furthermore, prolonged postoperative bowel paralysis, along with an anatomical predisposition to cecal volvulus due to a mobile cecum, could have caused the condition [7]. Cecal volvulus is a very rare postoperative complication but should be considered in cases of postoperative obstruction, especially after surgeries disrupting the fusion plane of the cecum and right colon.

## Conclusion

This is the first case report on cecal volvulus after open right hepatectomy. In our case, the combination of right-sided liver surgery, a highly mobile cecum, and prolonged paralysis could have led to cecal volvulus. Urgent CT scanning is necessary upon suspicion, with early surgical intervention being of utmost importance for the patient’s outcome.
